# Feasibility for Measuring Transverse Area Ratios and Asymmetry of Lumbosacral Region Paraspinal Muscles in Working Dogs Using Computed Tomography

**DOI:** 10.3389/fvets.2016.00034

**Published:** 2016-05-12

**Authors:** Bethany Cain, Jeryl C. Jones, Ida Holásková, Larry Freeman, Bess Pierce

**Affiliations:** ^1^Division of Animal and Nutritional Sciences, Davis College of Agriculture, Natural Resources, and Design, West Virginia University, Morgantown, WV, USA; ^2^Office of Statistics, Davis College of Agriculture, Natural Resources, and Design, West Virginia University, Morgantown, WV, USA; ^3^Department of Biomedical Sciences and Pathobiology, Virginia-Maryland College of Veterinary Medicine, Virginia Polytechnic Institute and State University, Blacksburg, VA, USA; ^4^Department of Population and Health Science, Virginia-Maryland College of Veterinary Medicine, Virginia Polytechnic Institute and State University, Blacksburg, VA, USA

**Keywords:** CT, core muscle, canine, lower back, cauda equina

## Abstract

**Objectives:**

Describe computed tomographic (CT) anatomy of canine lumbosacral (LS) paraspinal muscles, a method for measuring paraspinal muscle transverse area ratios and asymmetry using CT, and application of this method in a small sample of working dogs with versus without LS pain.

**Methods:**

Published anatomy references and atlases were reviewed and discrepancies were resolved by examination of anatomic specimens and multiplanar reformatted images to describe transverse CT anatomy of LS region paraspinal muscles. Sixteen Belgian malinois military working dogs were retrospectively recruited and assigned to LS pain positive versus negative groups based on medical record entries. A single observer unaware of dog group measured CT transverse areas of paraspinal muscles and adjacent vertebral bodies, in triplicate, for L5–S1 vertebral locations. A statistician compared muscle transverse area ratios and asymmetry at each vertebral location between groups.

**Results:**

The relative coefficient of variation for triplicate CT area measurements averaged 2.15% (*N* = 16). Multifidus lumborum (L6–7), psoas/iliopsoas (L5–6, L6–7), and sacrocaudalis dorsalis lateralis (L6–7, L7–S1) transverse area ratios were significantly smaller in dogs with LS pain (*n* = 11) versus without LS pain (*n* = 5) (*p* ≤ 0.05). Muscle asymmetry values were not significantly greater in dogs with versus without LS pain.

**Clinical relevance:**

Computed tomographic morphometry of LS region paraspinal muscles is a feasible objective method for use in future evidence-based research studies in working dogs. Potential future research applications include determining whether decreased paraspinal muscle area ratios and/or increased paraspinal muscle asymmetry could be used as markers for preclinical LS pain in stoic dogs or risk factors for other injuries in high performance canine athletes, or determining whether core muscle strengthening exercise prescriptions for dogs with LS pain have an effect on paraspinal muscle area ratios and asymmetry.

## Introduction

Lower back [lumbosacral (LS)] pain (LBP) is an important cause of debilitation and early retirement in working dogs (1–3). The standard diagnostic test is clinical detection of a painful reaction to palpation of the LS junction and/or dorsal extension of the tail (tail jack). For stoic, high drive, or aggressive working dogs, clinical detection of LBP may be difficult to demonstrate. For these dogs, diagnosis of LBP may be based on observed performance deficits, such as altered LS region posture during working tasks, reluctance to perform tasks requiring hyperextension of the LS spine, and/or altered movement of the tail (4). Commonly reported causes of LBP in dogs have included degenerative LS stenosis/disk disease, sacroiliac degenerative joint disease, and/or soft tissue injury in the LS region (1–6). Human and canine studies have indicated that chronic LBP often leads to maladaptive patterns of movement and abnormal resultant ground reaction forces, which may put patients at increased risk for injury and chronic, referred pain syndromes (7–9). In order to minimize risks of these complications, core muscle-strengthening and conditioning exercise prescriptions are increasingly being recommended and implemented for preventing or treating LBP in canine athletes (10–14) However, there are few evidence-based research studies supporting these prescriptions. A non-invasive, repeatable technique for objectively quantifying characteristics of LS region paraspinal muscles would be helpful for supporting development of these evidence-based research studies.

The anatomy and functions of canine LS region paraspinal muscles have been described in standard anatomic reference textbooks (15, 16). The lumbar epaxial spinal muscles include the following (from medial-to-lateral and dorsal to the level of transverse processes): multifidus lumborum (ML), longissimus lumborum (LL), and iliocostalis (IC) lumborum. All three of the epaxial spinal muscle systems serve, bilaterally, to extend the vertebral column. Unilaterally, they bend (flex) the column to that side such that the concavity of the bend faces to that side. The lumbar hypaxial muscles (medial-to-lateral and ventral to the level of transverse processes) include the following: psoas minor, psoas major [combines with the iliacus at the ventral ilium and becomes the iliopsoas (IP)], and the quadratus lumborum (QL). All these hypaxial muscles flex the lumbar portion of vertebral column and unilaterally serve to bend the column, so that the convexity of the bend faces to that side. The medial and lateral dorsal sacrocaudal muscles function bilaterally to extend/raise/lift the tail. If they contract unilaterally, they raise and deviate the tail toward the same side. The sacrocaudalis dorsalis medialis (SDM) is the continuation of the medial epaxial system, hence of the ML, into the sacral and tail region. It functions as the medial and short elevator of the tail in contrast to the lateral and long tail elevator, which is the sacrocaudalis dorsalis lateralis (SDL). Its cranial extent is its origin on the dorsolateral aspect of L7 vertebra. The SDL, the long elevator of the tail, is composed of muscle bundles that come together to form essentially the continuation of the LL into the sacral and tail region of the vertebral column. It originates *via* tendons from the first or second to seventh lumbar vertebrae as well as from the sacrum and tail vertebrae. Transverse sectional anatomy of canine lumbar and LS muscles has been described in veterinary anatomy atlases (17–20); however, identifications for some paraspinal muscles have been contradictory in these publications.

Computed tomography (CT) has been established as a method for quantifying cross-sectional area of lumbar region paraspinal muscles in humans with LBP (21–28). Similar intra- and inter-rater reliability has been reported for magnetic resonance imaging (MRI) and CT measures of paraspinal muscle cross-sectional areas in humans (24). A previous CT morphometry study of the canine vertebral canal in dogs with versus without cauda equina syndrome described the use of transverse vertebral canal area ratios calculated with area of the adjacent vertebral body as a correction factor for reducing variations due to differences in dog body sizes (29). A recently published study of canine paraspinal muscles in dogs with versus without degenerative LS stenosis described a MRI method for measuring transverse muscle area ratios and symmetry of the SDL, ML, and longissimus lumborum muscles at L7–S1 (30). Measurements of other muscles at other LS vertebral levels have not been reported in dogs.

We hypothesized that CT would be a feasible method for quantifying paraspinal muscle transverse area ratios and asymmetry in the canine LS region. Objectives of this pilot study were to describe (1) transverse CT anatomy of LS region (L5–S1) paraspinal muscles, (2) CT methods for measuring canine LS region paraspinal muscle transverse area ratios and asymmetry, and (3) application of these CT measurement methods in a small sample population of Belgian malinois military working dogs with versus without LBP.

## Materials and Methods

### Patient Selection Criteria

With hospital director approval, dogs were retrospectively recruited from medical record and computed tomographic (CT) image archives at the Daniel Holland Military Working Dog Veterinary Hospital at Lackland Air Force Base, TX, USA. All hospital requirements for ensuring confidentiality of patient data were maintained throughout the study. The search period for data retrieval was from April 2005 to July 2011. Inclusion criteria were as follows: Belgian malinois breed, CT scan that included the LS region, and available medical records describing clinical examination findings at the time of CT scanning. All CT images and medical records for dogs meeting these inclusion criteria were retrieved. A board-certified veterinary radiologist (Jeryl C. Jones) reviewed CT scans and excluded dogs if LS paraspinal muscles were not included in the scan field of view or if there was evidence of LS fractures, infection, neoplasia, or previous surgery.

### Transverse CT Anatomy Study

All digital CT images for included dogs were uploaded directly to a password-protected image analysis workstation (MacPro 12-core with 30″ Apple Cinema HD display, 1 Infinite Loop, Cupertino, CA, USA). Hard copy CT images for included dogs were first converted to DICOM format using a digital scanner system (Vidar Sierra Advantage, Sound Eklin, Carlsbad, CA, USA) and then transferred to the same image analysis workstation. All images reviews were performed using the same image analysis freeware (OsiriX version 4.1.2, http://www.osirix-viewer.com).

**Figure 1:**
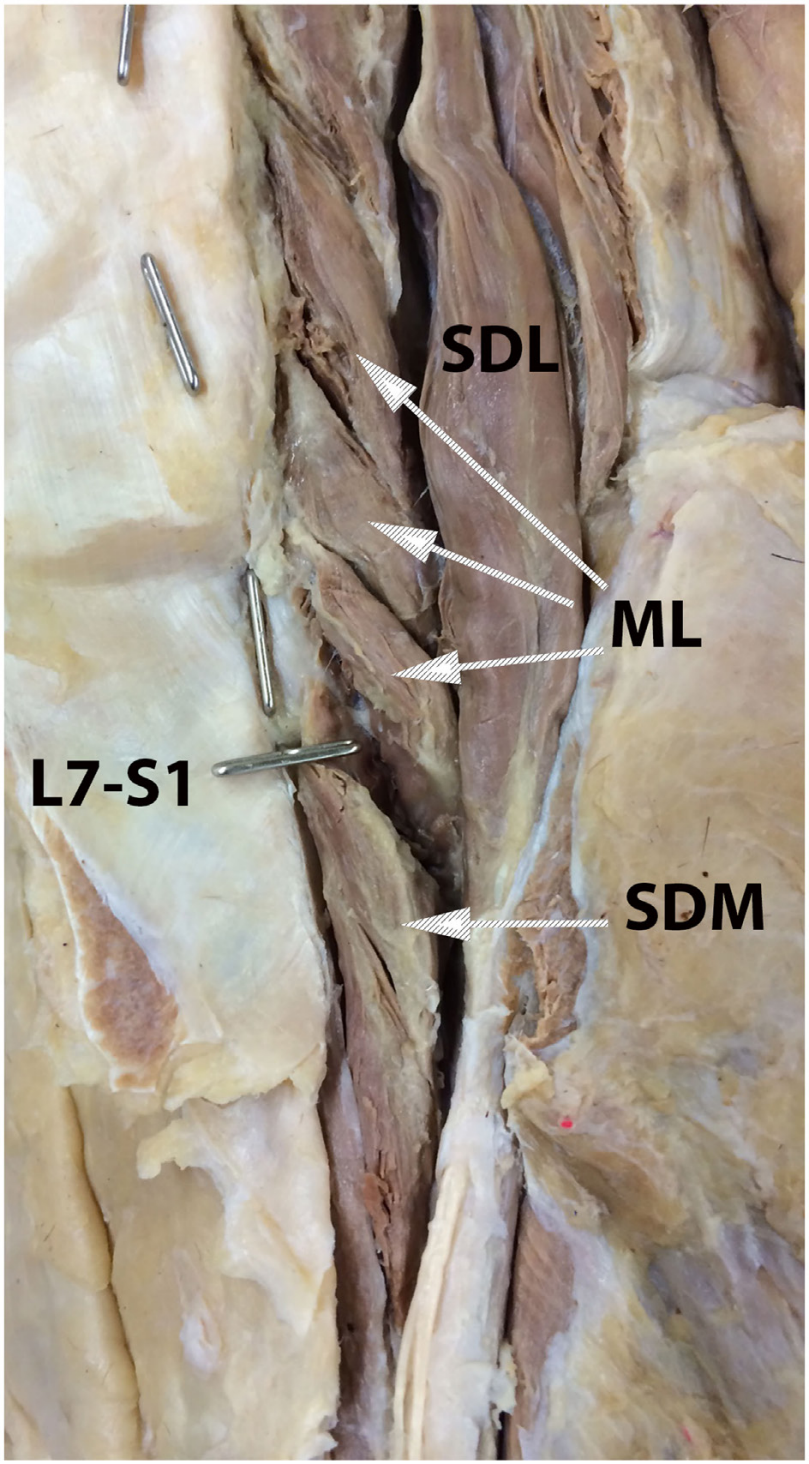
**Anatomic dissection photograph illustrating identification of the multifidus lumborum (ML), sacrocaudalis dorsalis medialis (SDM), and sacrocaudalis dorsalis lateralis (SDL) muscles**. Cranial is at the top of the image and the dog’s right is on the viewer’s right. Metal pins oriented in the sagittal plane mark the L7 and L6 spinous processes. The metal pin oriented in the transverse plane marks the L7–S1 junction. Attaching near the summit of the L7 spinous process is the SDM muscle bundle that continues caudally into the sacral region and on caudally, along with other muscle bundles into the tail region. This is the so-called short elevator of the tail. A longer muscle than is the SDM, the SDL originates as far craniad as from the cranial lumbar vertebrae and continues caudally into the sacral and tail regions. This muscle, which serves as the so-called long elevator of the tail, is readily dissectable as separate from the longissumus lumborum.

**Figure 2:**
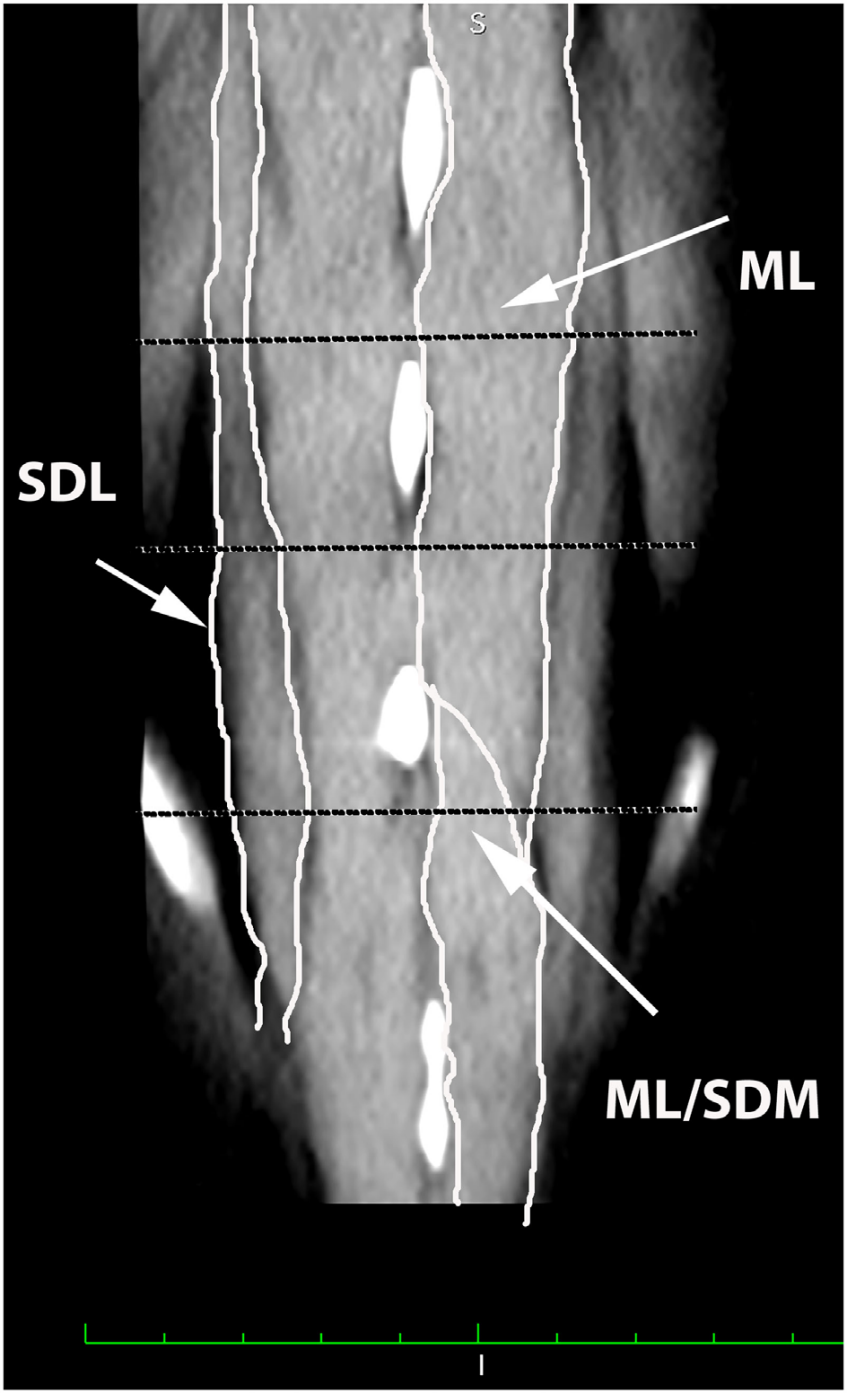
**Dorsal oblique multiplanar CT image at the level of the L5–S1 spinous processes, illustrating margins of the multifidus lumborum (ML), sacrocaudalis dorsalis lateralis (SDL), and sacrocaudalis dorsalis medialis (SDM)**. Cranial is at the top of the image and the patient’s right is on the viewer’s left. Transverse dotted lines illustrate the locations of L5–6, L6–7, and L7–S1.

**Figure 3:**
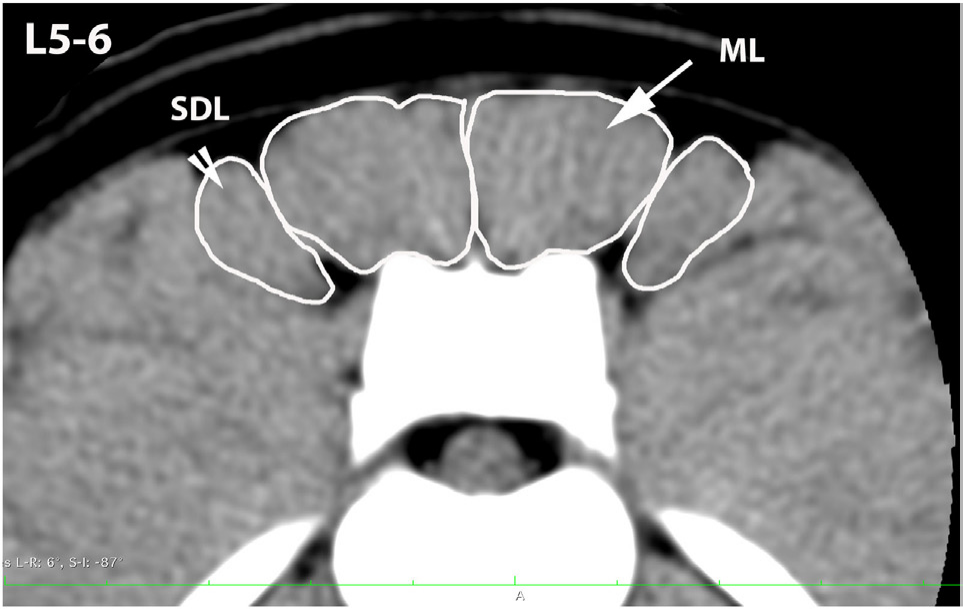
**Transverse CT image at the level of L5–6, illustrating margins of the multifidus lumborum (ML) and sacrocaudalis dorsalis lateralis (SDL)**.

**Figure 4:**
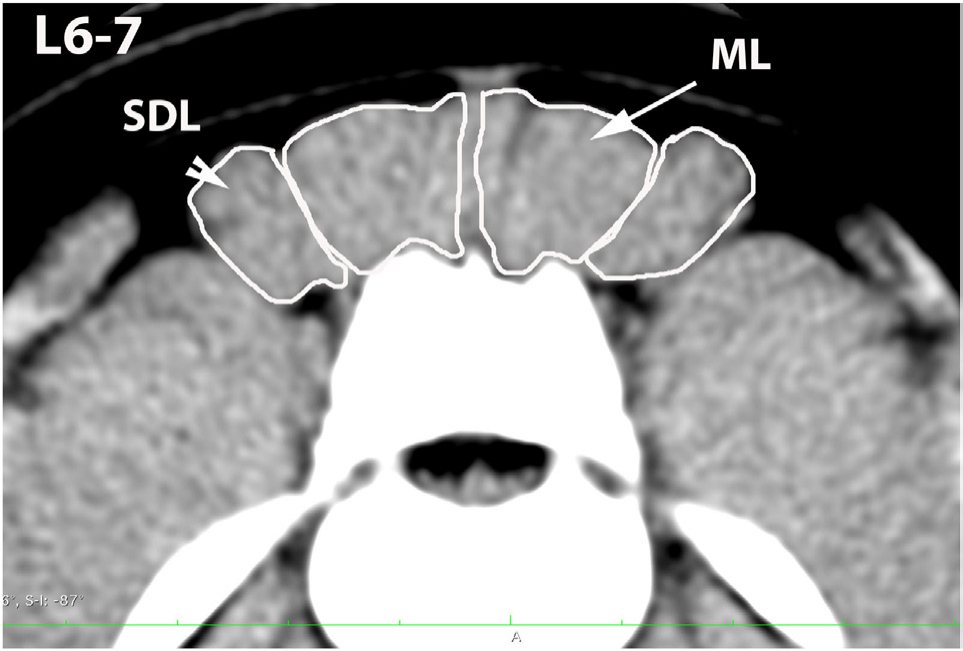
**Transverse CT image at the level of L6–7, illustrating margins of the multifidus lumborum (ML) and sacrocaudalis dorsalis lateralis (SDL)**.

**Figure 5:**
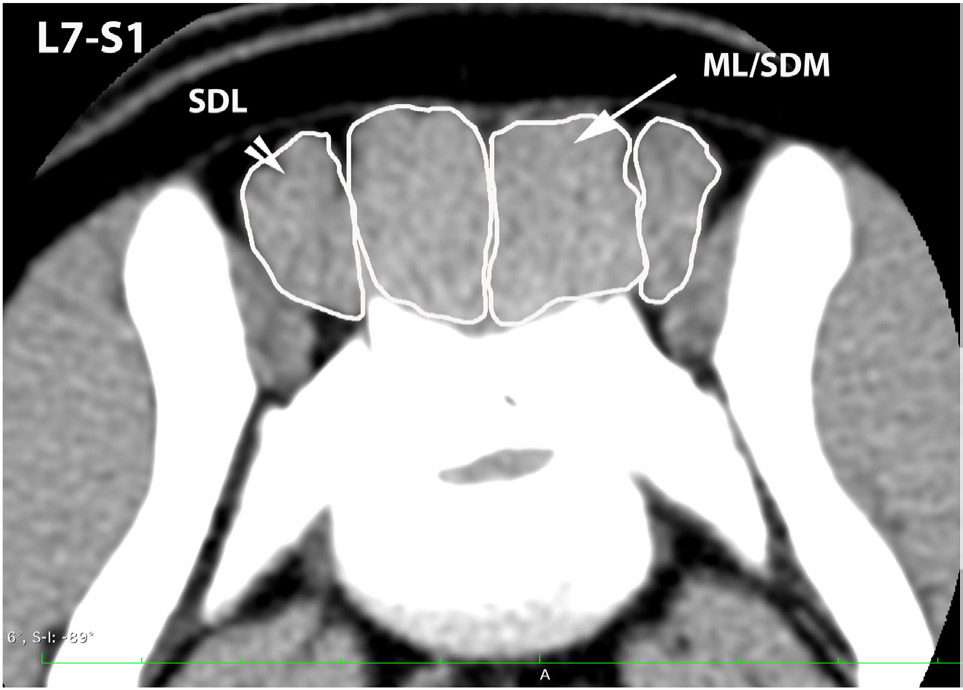
**Transverse CT image at the level of L7–S1, illustrating margins of the sacrocaudalis dorsalis lateralis (SDL) and the combined multifidus lumborum/sacrocaudalis dorsalis medialis (ML/SDM)**.

**Figure 6:**
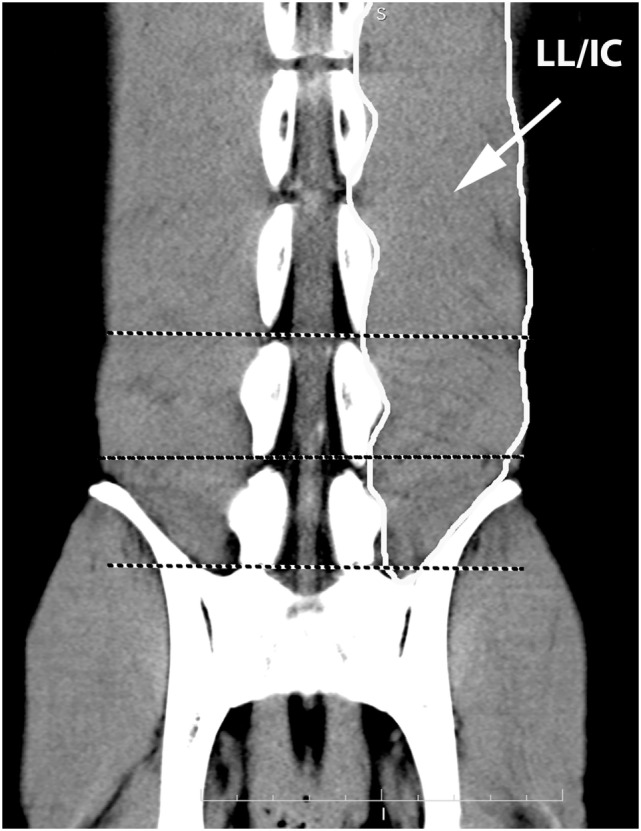
**Dorsal oblique multiplanar CT image at the level of the L5–S1 vertebral canal, illustrating margins of the combined longissimus lumborum/iliocostalis (LL/IC) muscle group**. Notice that this muscle group tapers at the level of L7–S1.

**Figure 7:**
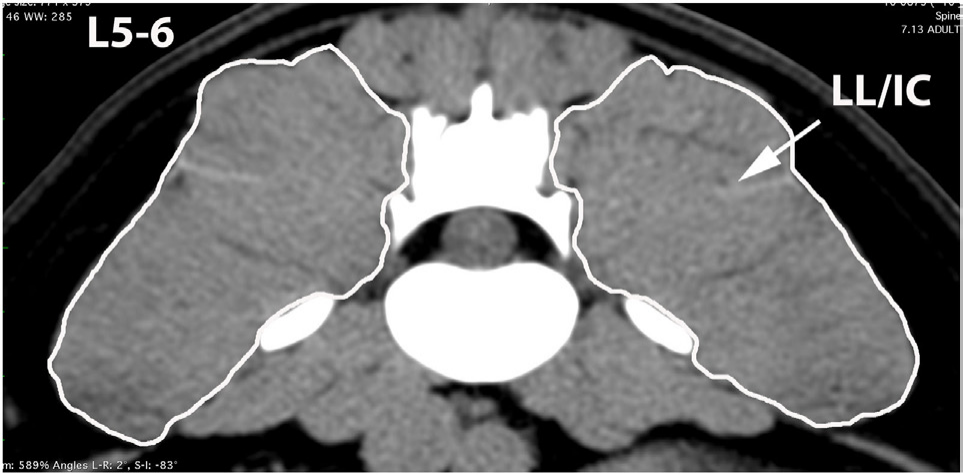
**Transverse CT image at the level of L5–6, illustrating margins of the combined longissimus lumborum/iliocostalis (LL/IC) muscle group**.

**Figure 8:**
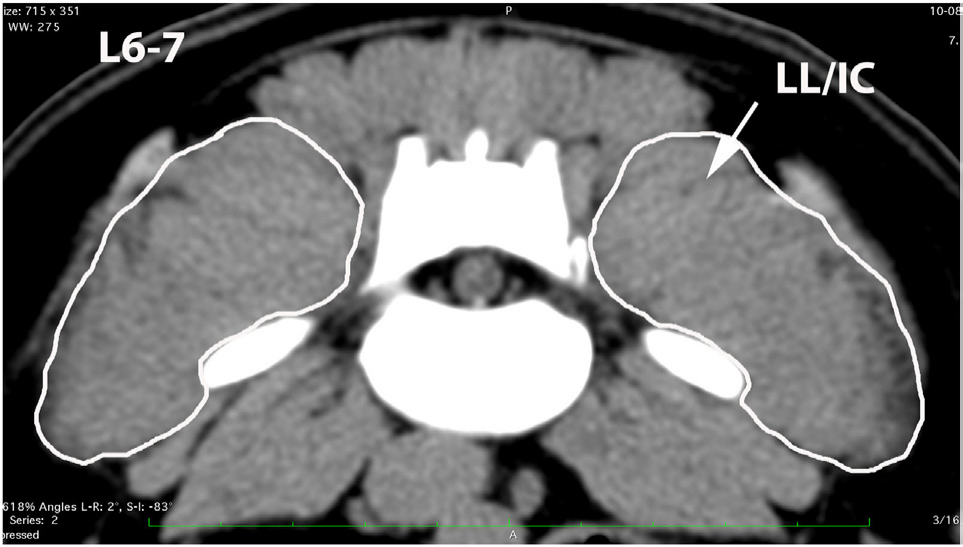
**Transverse CT image at the level of L6–7, illustrating margins of the combined longissimus lumborum/iliocostalis (LL/IC) muscle group**.

**Figure 9:**
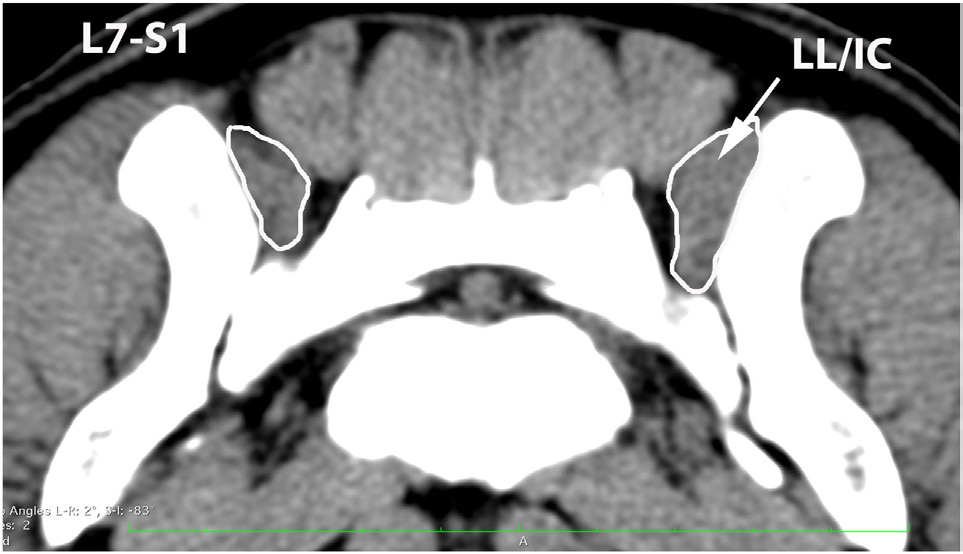
**Transverse CT image at the level of L7–S1, illustrating margins of the combined longissimus lumborum/iliocostalis (LL/IC) muscle group**.

**Figure 10:**
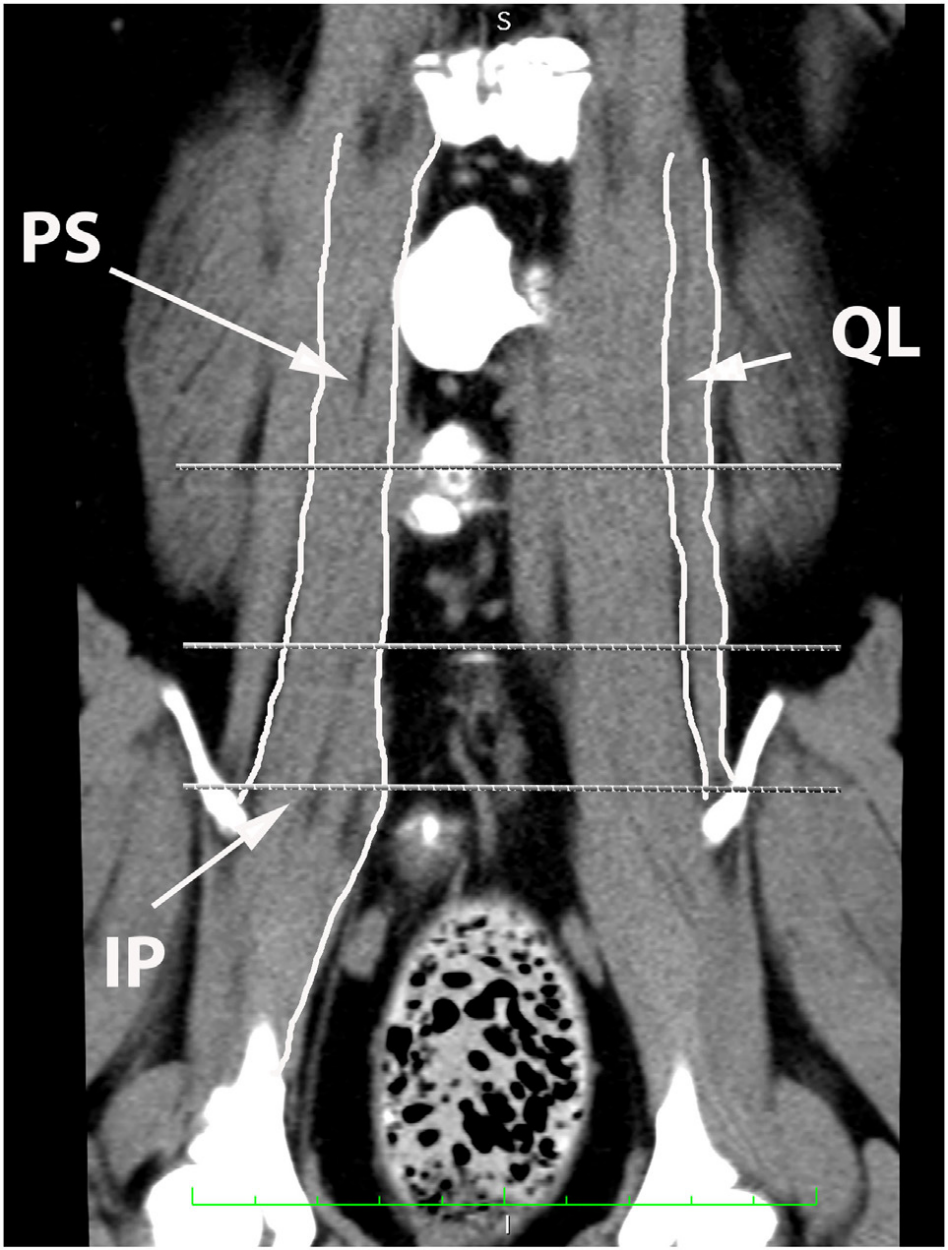
**Dorsal oblique multi-planar CT image at the level of the ventral vertebral bodies, illustrating margins of the quadratus lumborum (QL), psoas (PS), and iliopsoas (IP) muscles**.

**Figure 11:**
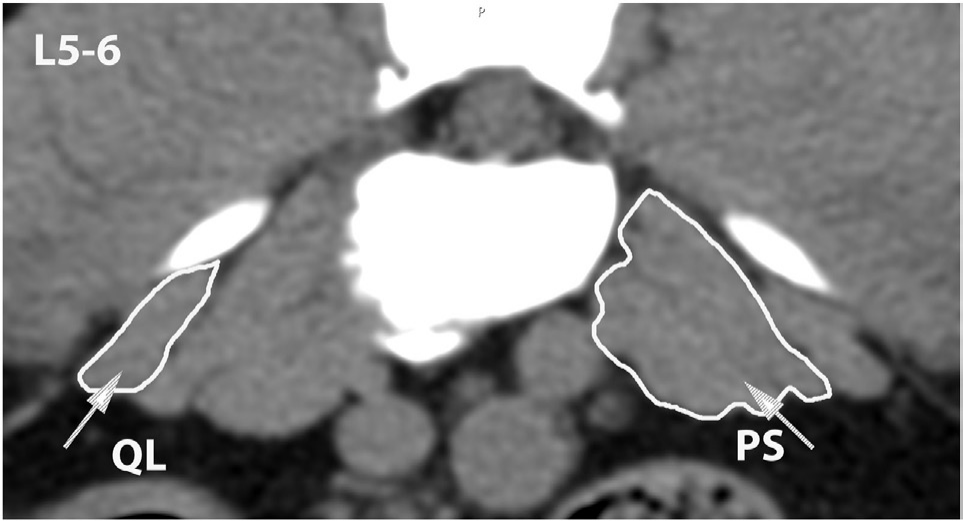
**Transverse CT image at the level of L5–6, illustrating margins of the quadratus lumborum (QL) and psoas (PS) muscles**.

**Figure 12:**
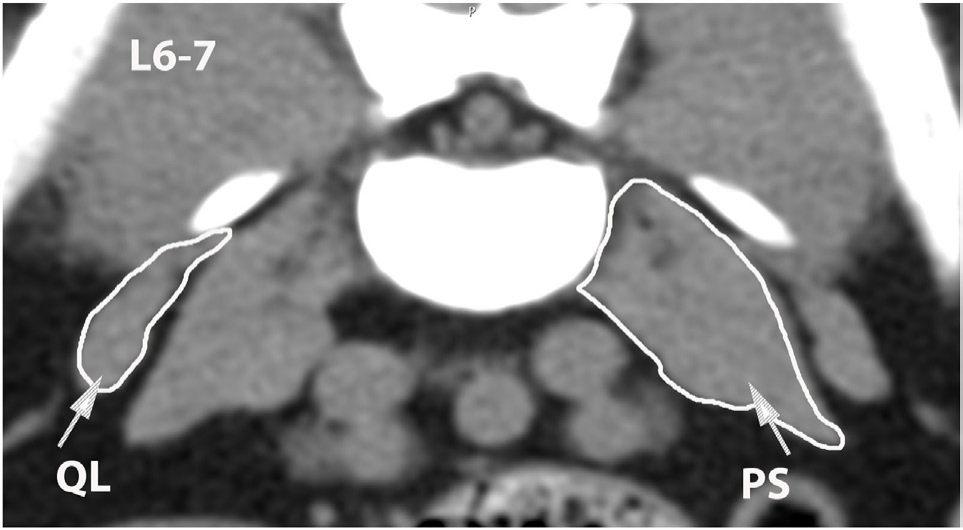
**Transverse CT image at the level of L6–7, illustrating margins of the quadratus lumborum (QL) and psoas (PS) muscles**.

**Figure 13:**
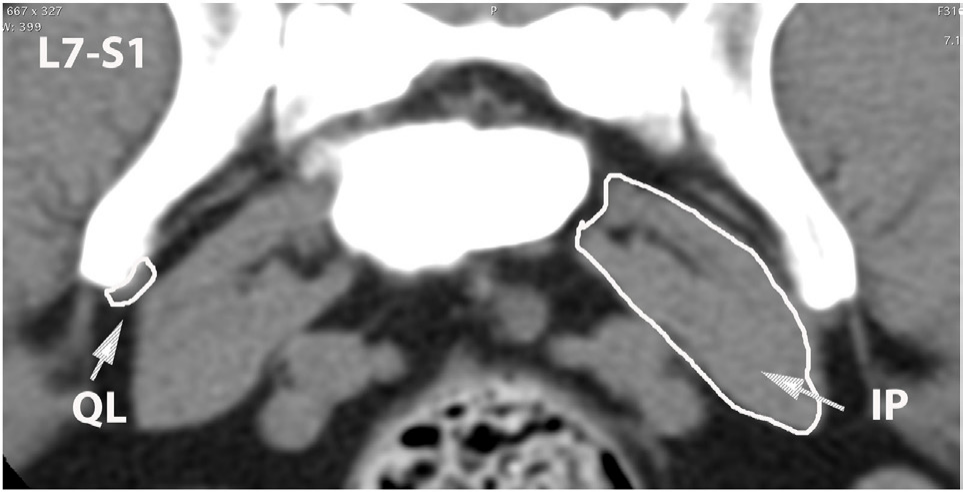
**Transverse CT image at the level of L7–S1, illustrating margins of the quadratus lumborum (QL) and iliopsoas (IP) muscles**.

A veterinary radiologist and veterinary anatomist reviewed anatomic reference textbooks and transverse sectional atlases and compared these to retrieved CT images (15–20). Discrepancies in transverse sectional atlas muscle identifications were resolved based on dissection of anatomic specimens (Figure 1), evaluation of multiplanar reformatted CT images, and a consensus agreement between both expert readers. For this study, the ML muscle was defined as the muscle lateral to the L5, L6, and cranial L7 spinous processes in transverse CT images (Figures 2–5). At the level of L7–S1, the muscle lateral to the caudal L7 spinous process was defined as a combination of ML and SDM. The SDL was defined as the muscle lateral to the ML and SDM. The combined LL/IC lumborum was defined as the muscle group lateral and ventral to the SDL (Figures 6–9). The QL was defined as the muscle lateral and ventral to the transverse processes of L5, L6, and L7, and that terminated on the medial margin of the ilium (Figures 10–13). The psoas was defined as the muscle medial to the QL and ventral to the L5, L6, and cranial L7 vertebral bodies. At the level of L7–S1, the muscle medial to the QL and ventral to the caudal L7 and cranial S1 vertebral bodies was identified as the IP (combined iliacus and psoas).

### Computed Tomographic Morphometry Technique

**Figure 14:**
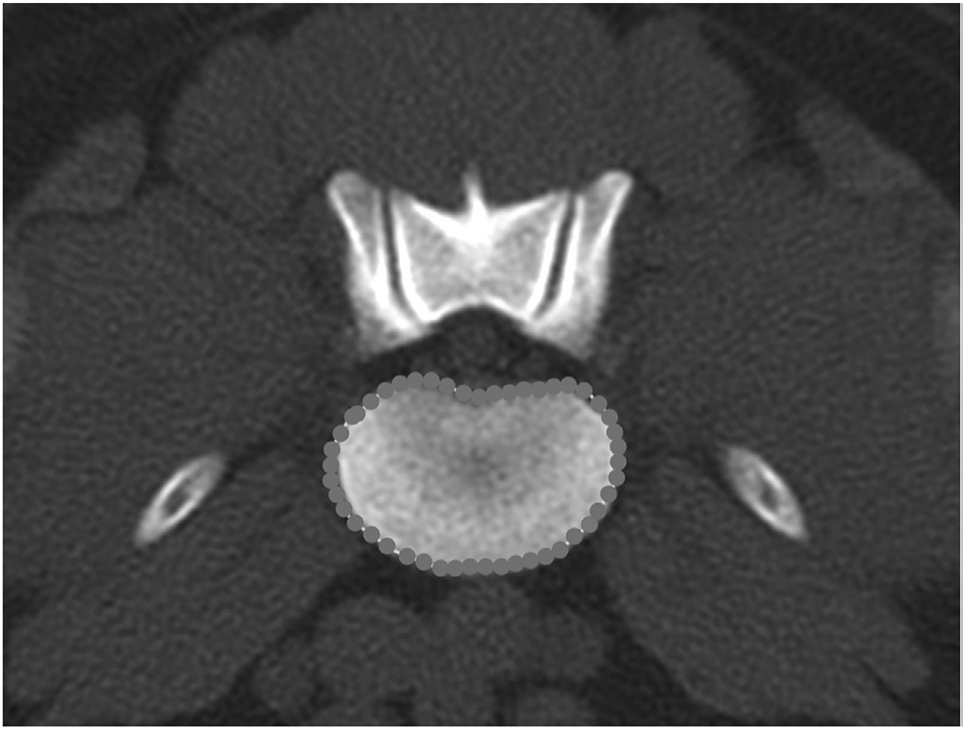
**Transverse bone window CT image at the level of L6–7, illustrating the hand-traced region of interest and calculated area value for the vertebral body**.

A single observer (Bethany Cain) performed all quantitative analyses of paraspinal muscles without knowledge of dog LS pain status. Centimeter scale tools in the image analysis freeware were used for calibration of area measurements in hard copy images. To perform the calibration for each scanned set of CT hard copy images, the observer first used the software’s line tool to mark locations of adjacent centimeter marks displayed in one of the image frames and the software automatically recorded this value as the number of pixels. The software’s centimeter scale tool was then used to assign that number of pixels the value of 1 cm. Once this calibration was performed, area measurements were converted from pixels to centimeters by the software program. The software’s “thick slab, mean” tool was used to standardize all transverse images to a 5-mm slice thickness before measurements were made. The observer used the image analysis freeware’s pencil tool to hand trace regions of interest (ROIs) around the outer margins of each of the paraspinal muscles defined by the anatomy study at the L5–6, L6–7, and L7–S1 vertebral levels. The slice location for measurements was chosen based on the transverse image that displayed the maximum height of the intervertebral foramen and complete caudal vertebral endplate margins. A standardized soft tissue window display setting (350 width, 40 level) was used for all muscle measurements. If the margins between adjacent muscles were not distinguishable, the observer extrapolated intermuscular margins by drawing a straight perpendicular line from the peripheral muscle margin to the adjacent vertebral margin. If the outer margin of a muscle was not completely included in the scan field of view, the muscle was excluded from the analyses. ROIs were also traced around vertebral bodies at the same locations as muscle ROIs and these were used as correction factors for variations in dog size (Figure 14). A standardized bone window display setting (1500 width, 300 level) was used for all vertebral body measurements. Areas for each muscle and adjacent vertebral body were measured in triplicate. After all ROI measurements were completed, mean area ratios for each muscle, each vertebral location, and each dog were calculated by the same observer using commercial software (Excel Office for Mac 2011, version 14.4.3) and the following formula (30):

$$\begin{array}{l} \text{Transverse area ratio }=\text{[(average of 3 right muscle area measurements}+\text{average of 3 left}\\ \text{muscle area measurements)/average of 3 vertebral endplate area measurements]} \end{array}$$

### Application of CT Morphometry Technique for Comparing Dogs with versus without Lumbosacral Pain

The same observer (Bethany Cain) reviewed medical record data after all CT mean area and area ratio calculations were completed. Dogs were assigned to the LS pain positive group if at least one of the following phrases was found in the medical record at the time the dog was presented for CT scanning: “pain/reaction on palpation of the LS junction,” “pain/reaction on elevation of the tail/tail jack,” or “LS hyperesthesia.” A statistician (Ida Holásková) selected and performed all statistical tests using commercial software (JMP^®^, Version Pro 11, SAS Institute, Inc., Cary, NC, USA, Copyright ©2013; SAS^®^, Version 9.3, SAS Institute, Inc., Cary, NC, USA, Copyright ©2002–2010). Intra-observer repeatability (relative coefficient of variation %, CV) for triplicate area measurements was calculated for each dog, each side, and each variable using the following formula:

CV=[(SD/mean)×100]

Each response variable was first tested for normality using the Shapiro–Wilk *W* test. For variables that were not normally distributed, a log 10 or a square root transformation was applied. Variables with fewer than two available values were excluded from the analyses. Muscle asymmetry values for each muscle and each vertebral location were calculated for each dog using the following formula (28):

$$\begin{array}{l} \text{Asymmetry value}=[\left(\text{Average of 3 right area measurements}\right)\;\\ -\;\left(\text{Average of 3 left area measurements}\right)] \end{array}$$

Analysis of covariance (ANCOVA) was performed on the area ratio and asymmetry, to adjust the effect of LS pain for the possible dog-specific covariates such as age, weight, and sex. To test the hypothesis that means muscle transverse area ratios would be smaller in dogs with LBP, a lower tail *t*-test was performed for normally distributed data. In order to control for the type I error rate, when analyzing 13 muscle areas simultaneously, the Benjamini–Hochberg adjustment with 15% false discovery rate was applied to *p* values obtained from the *t*-tests (31). To test the hypothesis that muscle asymmetry would be greater in dogs with LBP, the upper tail *t*-test was performed. Power analysis was done after the aforementioned statistical tests. For each test, and before adjustment, statistical significance was defined as *p* ≤ 0.05.

## Results

### Description of Sample Population

A total of 16 dogs met all inclusion criteria for the study. Eleven dogs were assigned to the LS pain positive group and five dogs were assigned to the LS pain negative group (Table 1). Dogs in the LS pain negative group had been presented for CT scanning for the following reasons: hindlimb lameness (*n* = 2) and another research project (*n* = 3). Eight digital and eight hard copy CT studies were used in the analyses. All dogs had been sedated or anesthetized and positioned in dorsal recumbency for scanning. All dogs were positioned with the LS spine in an extended position. All scans were acquired on site at the MWD hospital using multidetector CT scanners with a 512 × 512 matrix (HiSpeed Advanced System No. HSA2 or LightSpeed VCT, GE Medical Systems, Milwaukee, WI, USA). Other CT technical parameters had varied at the discretion of the veterinary radiologist overseeing the case.

**Table 1:** **Description of sample population of 16 Belgian malinois military working dogs included in the study**.

Characteristics		Lumbosacral pain positive (*n* **=** 11)	Lumbosacral pain negative (*n* **=** 5)
Sex	Female	2	2
	Male	9	3
Age (years)	Mean (SD)	6.5 (3.1)	5 (2.3)
	Median (range)	7 (2–11)	4 (3–8)
Weight (kg)	Mean (SD)	29.8 (4.7)	27.3 (3.8)
	Median (range)	29 (25–40)	27 (23–32)

*Age and weight data were normally distributed*.

**Figure 15:**
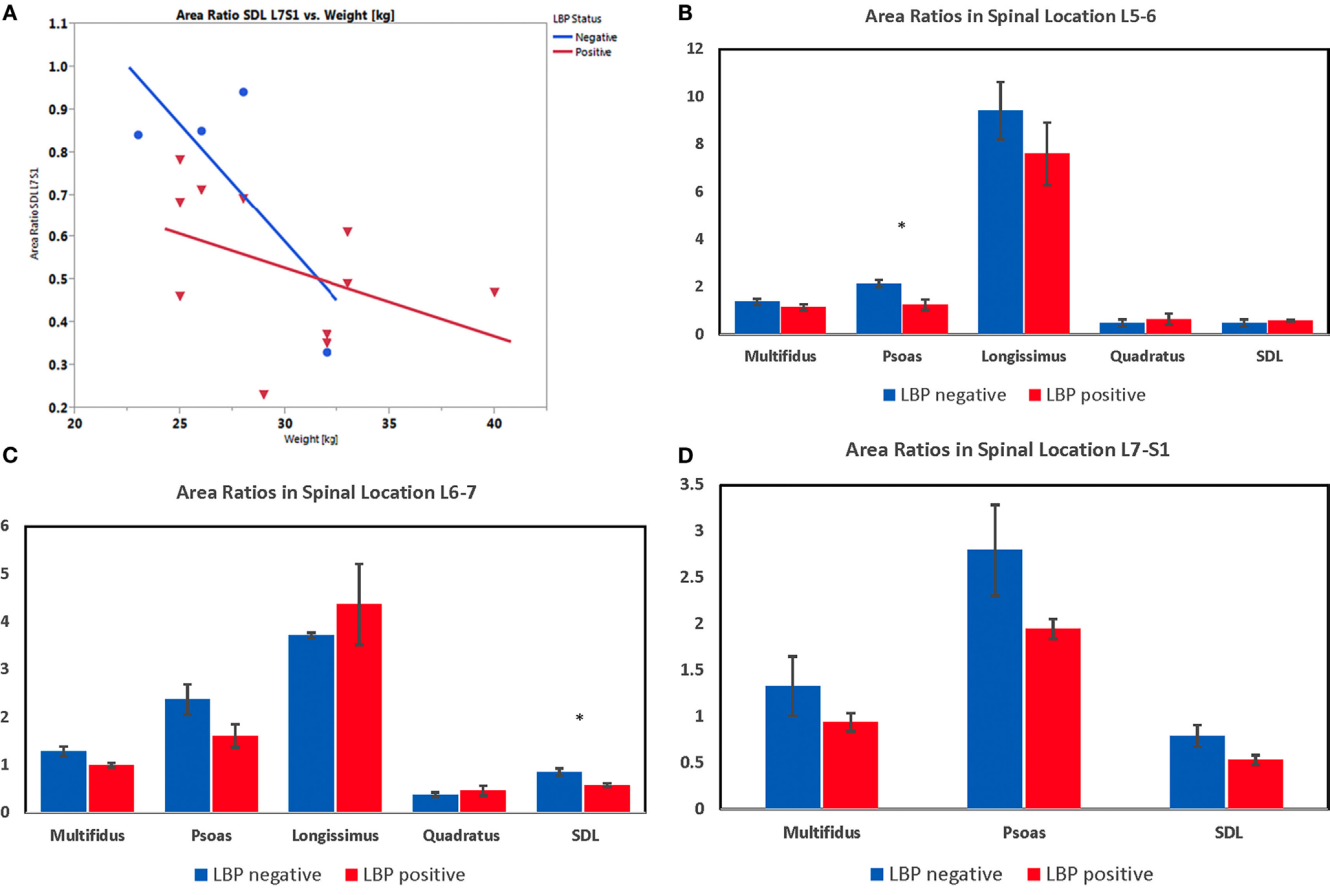
**(A)** Plot representing the ANCOVA depicting the effect of lower back pain (LBP) and weight (covariate) on transverse area ratio of the sacrocaudalis dorsalis lateralis muscle at L7–S1 in Belgian malinois working dogs (*n* = 15). One dog was excluded from these analyses, because the SDL was partially cut off in the scan field of view at L7–S1. Weight is a significant covariate (*p* = 0.029), and there was no significant interaction of LBP and weight on transverse area ratio. **(B)** Transverse area ratios of muscles measured at the level of the disc space between the fifth and sixth lumbar vertebrae. **(C)** Transverse area ratios of muscles measured at the level of the disc space between the sixth and seventh lumbar vertebrae. **(D)** Transverse area ratios of muscles measured at the level of the disc space between the seventh lumbar and first sacral vertebrae. (*indicates significant at *p* < 0.05 for the particular spinal location detected by lower-tail *t*-test after Benjamini–Hochberg adjustment.)

The relative coefficient of variation (CV, intra-observer repeatability) for all triplicate CT area measurements averaged 2.15% (range 0.7–4.3%). When calculated by dog group, the average CV for triplicate measures was 1.45% (0.57–2.82%) for the LS pain positive group and 2.85% (1.85–3.45) for the control group. Dogs’ age was a significant covariate in one of the 13 muscle areas (QL at L5–6) for both area ratio and asymmetry; however, the low sample size at this location (*n* = 7) lead to very low statistical power for the ANCOVA (<20%). Weight was also detected as significant covariate in one of the 13 muscle locations (SDL at L7–S1, Figure 15A), with negative slope (*p* = 0.03) and power of 67% for transverse area ratio. There was no significant interaction detected between LS pain and weight of dogs in this vertebral region. For asymmetry, dog’s weight was found as significant covariate in ML at L5–6 with significant interaction of body weight and LS pain (*p* = 0.018). However, the power of this test was only 25% (data not shown).

Results from the one-tailed *t*-test after Benjamini–Hochberg adjustment indicated that dogs with LS pain had significantly smaller transverse area ratios for the following muscles and locations: psoas at L5–6 (*p* = 0.007) and L6–7 (*p* = 0.049; Figures 15B,C), ML at L6–7 (*p* = 0.025; Figure 15C), and SDL at L6–7 (*p* = 0.012) and L7–S1 (*p* = 0.035; Figures 15C,D). There were no significant differences detected in mean transverse area ratios for longissimus and quadratus muscles. Results from the one-tailed *t*-test and Benjamini–Hochberg adjustment indicated that paraspinal muscle asymmetry was not significantly greater in any of the muscle areas in dogs with versus without LS pain. However, with a large variability in asymmetry data, the power of the *t*-test was <31% for all muscle areas.

## Discussion

The intention of the current pilot study was to develop and describe an objective method for quantifying LS paraspinal muscles in working dogs, with the long-term goal of supporting evidence-based research studies. Application of the method was illustrated in a small sample of working dogs with versus without clinically detected LS pain. Utility of these measures as a diagnostic tool for individual patients was not tested. Findings indicated that CT is a feasible method for measuring LS paraspinal muscle transverse area ratios and asymmetry in groups of dogs for research purposes. The use of multiplanar reformatting and anatomic dissections was helpful for clarifying muscle anatomy in transverse CT images. Inclusion of L5–6 and L6–7 in the measurements allowed detection of muscle area differences that would have been missed if only the L7–S1 level was measured. Paraspinal muscles measured at the L5–6 and L6–7 vertebral levels were those primarily responsible for flexion, extension, and lateral movements of the caudal lumbar spine. Paraspinal muscles measured at L7–S1 also included those responsible for movement of the tail and rear limbs.

Vertebral body transverse area measurements were used as correction factors for muscle transverse area measurements in order to minimize effects of dog body size variations for group comparisons. Intra-observer repeatability for muscle and vertebral body measurements was high for dogs in both LS pain positive and negative groups. We identified the evidence that dog weight or age were possibly covariates for transverse area ratios or asymmetry in some muscle areas. However, for age covariate analyses, the power of the tests was low due to the small sample size. The negative slope between the transverse area ratio and weight indicated that the area ratio may decrease with increasing weight regardless of the pain category, but more dogs over 35 kg without pain should be included in the analysis in order to more definitively test this theory. Significant differences in transverse area ratios were identified for groups of dogs with versus without LS pain. Results of comparisons were consistent with those reported in previous CT morphometry studies of humans with versus without LBP (22, 26, 27), and a previous MRI morphometry study of dogs with versus without degenerative LS stenosis (30). Therefore, either MRI or CT could be used for measuring muscles in future research studies and the selection of modality could be based on availability and cost.

Limitations of the current study included a small sample size, mixture of digital and hard copy CT images, and variable CT technical parameters. We attempted to minimize outside sources of measurement variation by standardizing the CT image analysis workstation/software, CT slice thickness, and window/level display settings; and using an average of triplicate area measures for group comparisons. Authors acknowledge that there was a sample population bias for this study in that only Belgian malinois military working dogs presenting to a tertiary referral MWD hospital for CT scans that included the LS region were sampled. Whether the findings from this study would be generalizable for other dog breeds and for non-working dogs, therefore, remains unknown. Belgian malinois were chosen for the study because they are one of the most commonly used breeds for military service.

Potential future research applications for methods described in the current pilot study could include determining whether decreased paraspinal muscle area ratios and/or increased paraspinal muscle asymmetry could be used as markers for preclinical LS pain in stoic dogs or risk factors for other injuries in high performance canine athletes. Another potential research application could include determining whether core muscle strengthening exercise prescriptions for dogs with LS pain have an effect on paraspinal muscle area ratios and asymmetry. Effects of other possible factors for decreased muscle transverse area ratios and increased asymmetry, such as positioning variation, observer expertise for determining pain status, presence of concurrent diseases, prior or ongoing use of medications, duration of signs, sex, and type of work, may also warrant further investigation.

In conclusion, findings from the current pilot study indicated that CT measurements of transverse area ratios and asymmetry are feasible methods for objective, quantitative characterization of LS region paraspinal muscles for use in future canine research studies. Additional studies are needed to test the effects of other clinical factors on muscle quantitative characteristics.

## Ethics Statement

All dogs were owned by the Department of Defense, and use of the archived medical record data was approved by the Director of the Military Working Dog hospital.

## Author Contributions

BC contributed all of the recorded data for the paper, in addition to meeting all four other ICJME requirements. JJ served as the mentoring author for BC and contributed all of the veterinary radiology content for the paper, in addition to meeting all four other ICJME requirements. IH contributed all of the statistical analysis content for the paper, in addition to meeting all four other ICJME requirements. LF contributed all of the veterinary anatomy content for the paper, in addition to meeting all four other ICJME requirements. BP contributed all of the veterinary sports medicine and rehabilitation content for the paper, in addition to meeting all four other ICJME requirements.

## Conflict of Interest Statement

The authors declare that the research was conducted in the absence of any commercial or financial relationships that could be construed as a potential conflict of interest.
